# Remimazolam Bolus Prevents Emergence Agitation After Rhinologic Surgery: A Randomized, Triple-Blind, Controlled Trial

**DOI:** 10.3390/medsci14010129

**Published:** 2026-03-10

**Authors:** Grgur Prižmić, Filip Periš, Marinela Jozeljić Pešić, Ana Maria Mitar, Ana Bego, Sanja Pavičić Perković, Sanda Stojanović Stipić

**Affiliations:** 1 Department of Anaesthesiology, Division of Anaesthesiology, University Hospital of Split, 21000 Split, Croatia; grgur.prizmic@kbsplit.hr (G.P.); filip.peris@kbsplit.hr (F.P.); marinela.jozeljic.pesic@kbsplit.hr (M.J.P.); ana.bego@kbsplit.hr (A.B.); sanja.pavicic.perkovic@kbsplit.hr (S.P.P.); sanda.stojanovic.stipic@kbsplit.hr (S.S.S.); 2 School of Medicine, University of Split, 21000 Split, Croatia

**Keywords:** remimazolam, emergence agitation, rhinologic surgery, sevoflurane, randomized controlled trial, post-anesthesia care unit

## Abstract

**Background/Objectives:** Emergence agitation (EA) is common after rhinologic surgery and may cause self-injury, bleeding, and prolonged post-anesthesia care unit (PACU) stay. Remimazolam is an ultra-short-acting benzodiazepine that may reduce EA without delaying recovery. The objective of this study was to evaluate the effect of a single dose of remimazolam administered at the end of surgery on the incidence of EA in adult patients undergoing nasal surgery. **Methods:** In this prospective, randomized, triple-blind, placebo-controlled trial, 62 adults undergoing elective rhinologic surgery under sevoflurane anesthesia received either remimazolam 0.1 mg/kg or saline immediately after sevoflurane discontinuation and before extubation. EA was assessed using the Richmond Agitation–Sedation Scale (RASS) at extubation and every 5 min for 30 min in the PACU. The primary outcome was presence of EA (RASS ≥ 2) at extubation. Secondary outcomes included Aldrete recovery scores, VAS, PONV incidence and safety outcomes. The study was registered at ClinicalTrials.gov (NCT06398275; 3 May 2024). **Results:** EA occurred in 12/32 patients (37.5%) in the control group and 0/30 (0%) in the remimazolam group (*p* < 0.001). Extubation time and operative durations were similar between groups. More patients in the remimazolam group achieved an Aldrete score ≥ 9 at extubation (76.7% vs. 50.0%, *p* = 0.030). Severe agitation (RASS ≥ 3) requiring rescue sedation occurred in 6/32 control-group patients and in 0/30 patients in the remimazolam group (*p* = 0.025). Pain scores were low (no VAS > 2). PONV occurred in one patient per group. Clinically relevant postoperative nasal bleeding requiring intervention occurred in 2/32 control-group patients and in 0/30 remimazolam-group patients. No laryngospasm or respiratory complications within 24 h were observed. **Conclusions:** A single remimazolam bolus given at the end of surgery prevented clinically relevant EA after rhinologic surgery without delaying early recovery.

## 1. Introduction

Emergence agitation (EA) is a behavioral disturbance that may occur during early recovery from general anesthesia [1,2]. Clinically, it most often presents with restlessness, disorientation, and purposeless movements; in more severe cases, patients may become combative [2,3]. Although EA is often transient, it is particularly relevant after rhinologic surgery, where uncontrolled movements may trigger bleeding, cause displacement of nasal packing, lead to accidental removal of intravenous lines, and prolong post-anesthesia care unit (PACU) stay and monitoring [1,2,3,4].

Reported rates of EA in adults vary widely across surgical procedures, anesthetic techniques, and assessment methods, ranging from 3% to more than 20% [1,4]. In the specific setting of nasal surgery, EA has been reported in approximately 20–30% of adult patients [2,5,6]. Importantly, EA in this population is not only disruptive but also associated with higher rates of early postoperative events, including cough, desaturation, breath-holding, laryngospasm, and nasal bleeding [6].

Several pharmacologic approaches have been evaluated for EA prevention, including opioids (e.g., fentanyl/remifentanil or butorphanol), benzodiazepines, α2-adrenergic agonists (e.g., dexmedetomidine), non-opioid analgesics and NMDA-receptor antagonists such as ketamine. While several of these strategies reduce agitation, they may also come at the cost of delayed recovery, respiratory depression, or hemodynamic instability [7,8,9].

Remimazolam is an ultra-short-acting benzodiazepine metabolized by tissue esterases, with rapid onset and offset and minimal accumulation [10,11]. This profile makes it an attractive candidate for targeting the brief but high-risk emergence period without compromising early recovery. Remimazolam has been shown to reduce emergence agitation in pediatric patients under sevoflurane anesthesia [12], and recent randomized trials in adults undergoing nasal surgery have also reported reduced agitation using remimazolam-based approaches [13,14,15].

The objective of this study was to evaluate the effect of a single dose of remimazolam administered at the end of surgery on the incidence of EA in adult patients undergoing nasal surgery.

## 2. Materials and Methods

### 2.1. Study Design, Ethics, and Registration

This prospective, randomized, triple-blind, placebo-controlled trial was conducted at the Department of Anaesthesiology, University Hospital of Split, Croatia. The study protocol was approved by the institutional Ethics Committee (520-03/24-01/13) and was registered at ClinicalTrials.gov (NCT06398275) on 3 May 2024 before patient enrollment. Written informed consent was obtained from all participants. The study was conducted and reported in accordance with the CONSORT guidelines.

### 2.2. Participants

Adults aged 18–65 years, American Society of Anesthesiologists (ASA) physical status I–II scheduled for elective rhinologic surgery under general anesthesia were eligible. Exclusion criteria were known hypersensitivity to remimazolam or other benzodiazepines, treatment with psychiatric medications within 6 months before surgery, and pregnancy.

**Figure 1:**
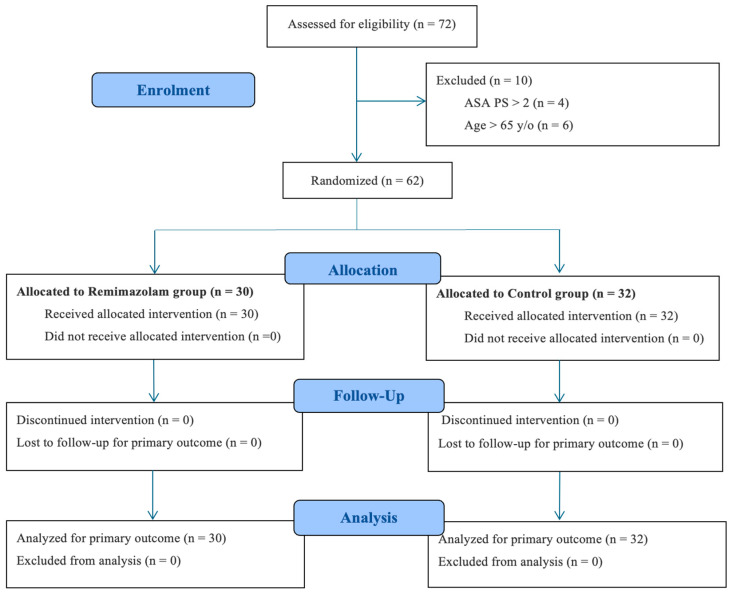
Consolidated Standards of Reporting Trials (CONSORT) diagram. ASA PS: American Society of Anesthesiologists physical status.

Between March 2024 and February 2025, 72 patients were assessed for eligibility. Ten patients were excluded (ASA physical status > II, *n* = 4; age > 65 years, *n* = 6). A total of 62 patients were enrolled and randomized to receive remimazolam (*n* = 30) or placebo (*n* = 32). All randomized participants received the allocated intervention, and no patients were lost to follow-up (Figure 1).

### 2.3. Randomization and Blinding

Randomization was performed using a computer-generated simple randomization sequence with an intended 1:1 allocation ratio and applied sequentially in the order of enrollment. The randomization sequence was generated by an investigator not involved in patient recruitment, intraoperative management, extubation, postoperative assessment, or data analysis.

Allocation concealment was ensured by preparation of the study medication by an anesthesiologist not involved in intraoperative management, extubation, postoperative assessment, or data analysis. Remimazolam and saline are both clear, colorless solutions and were drawn into identical syringes with equal volume and indistinguishable appearance, and were labeled with the study ID only. During preparation and administration, no differences in viscosity or syringe plunger resistance were noted by the anesthesiologist preparing or administering the study medication.

Patients, care providers, the PACU outcome assessor, and the investigator performing statistical analysis remained blinded to group allocation until completion of the analysis.

### 2.4. Anesthesia Protocol and Intervention

All patients received a standardized anesthetic protocol. Induction was performed with intravenous fentanyl 2 µg/kg, propofol 2 mg/kg, and rocuronium 0.6 mg/kg. Anesthesia was maintained with sevoflurane in an oxygen/air mixture and titrated to maintain a bispectral index (BIS) between 40 and 60.

Mechanical ventilation was volume-controlled with a tidal volume of 6–8 mL/kg and a respiratory rate of 10–12 breaths/min. An additional 1–3 µg/kg of fentanyl was administered before the start of surgery, depending on the type of procedure. Supplemental doses of fentanyl and rocuronium were administered only in patients undergoing rhinoseptoplasty or combined procedures.

At the beginning of surgery, all patients received dexamethasone 0.1 mg/kg intravenously. Shortly before the end of surgery, ketoprofen 100 mg and granisetron 1 mg were administered intravenously.

At the end of surgery, immediately after discontinuation of sevoflurane and before extubation, patients received either remimazolam 0.1 mg/kg or an equal volume of normal saline as a single intravenous bolus. Extubation was performed after return of spontaneous ventilation and adequate responsiveness to verbal commands.

Rescue sedation was planned for severe agitation (RASS ≥ 3) using a propofol bolus of 0.5 mg/kg.

### 2.5. Outcomes

Baseline demographic and clinical data were collected, including age, sex, height, weight, smoking status, and chronic comorbidities.

Emergence agitation was evaluated using the Richmond Agitation–Sedation Scale (RASS), a 10-point scale ranging from −5 (unarousable) to +4 (combative). The primary outcome was defined as clinically relevant agitation, indicated by a RASS score ≥ 2 immediately after extubation.

Secondary RASS assessments were performed every 5 min for 30 min in the post-anesthesia care unit (PACU), yielding a total of seven assessments per patient. Pain intensity was assessed using a visual analog scale (VAS; 0 = no pain, 10 = worst imaginable pain) and recorded at the same time points as RASS assessments. Postoperative nausea and vomiting (PONV) were evaluated at each PACU time point. Early recovery was assessed using the Aldrete score.

### 2.6. Sample Size

Sample size was calculated a priori based on pilot data (*n* = 16). To detect a clinically relevant difference in the primary outcome with 80% power and a two-sided alpha of 0.05, a minimum of 58 patients was required. To account for variability, the planned sample size was 60 patients. Sixty-two patients were ultimately enrolled due to the structure of the randomization list.

### 2.7. Statistical Analysis

Statistical analyses were performed using MedCalc Statistical Software version 22.014 (MedCalc Software Ltd., Ostend, Belgium). Data distribution was assessed using the Shapiro–Wilk test. Continuous variables are presented as mean ± SD for normally distributed data or as median (Q1, Q3) for non-normally distributed data, and were compared using the independent t-test or the Mann–Whitney U test, as appropriate. Categorical variables are presented as numbers (%) and were compared using the chi-square test or Fisher’s exact test. All statistical tests were two-tailed, and *p* < 0.05 was considered statistically significant. For the primary outcome, absolute risk reduction (ARR) with 95% confidence intervals and the number needed to treat (NNT) were calculated.

## 3. Results

A total of 62 patients were randomized and included in the final analysis (remimazolam group, *n* = 30; control group, *n* = 32). No participants were lost to follow-up, and no protocol deviations occurred (Figure 1).

Baseline demographic and clinical characteristics were comparable between groups (Table 1), with no statistically significant differences in age, sex, ASA physical status, BMI, smoking status, or comorbidities.

The distribution of surgical procedures was also similar between groups, with septoplasty, rhinoseptoplasty, and functional endoscopic sinus surgery (FESS) being the most common interventions (Table 2).

### 3.1. Emergence Agitation

Clinically relevant emergence agitation, defined as RASS ≥ 2 immediately after extubation, occurred in 12 of 32 patients (37.5%) in the control group and in 0 of 30 patients (0%) in the remimazolam group (*p* < 0.001, Fisher’s exact test). The absolute risk reduction was 37.5 percentage points (95% CI 11.6 to 54.7), corresponding to a number needed to treat of 3. No patient in either group developed deep sedation during PACU observation (RASS ≤ −3).

Severe agitation (RASS ≥ 3), requiring rescue sedation per protocol, occurred in 6/32 patients (18.8%) in the control group and in 0/30 patients (0%) in the remimazolam group (*p* = 0.025). All six affected control-group patients received rescue propofol, whereas no patient in the remimazolam group required additional sedation.

### 3.2. Intraoperative and Emergence Time Variables

Durations of surgery, anesthesia, and time to extubation were similar between groups (Table 3). No clinically meaningful prolongation of emergence was observed in the remimazolam group.

### 3.3. Early Recovery

Recovery profiles were favorable in both groups. A higher proportion of patients in the remimazolam group achieved an Aldrete score ≥ 9 immediately after extubation compared with controls (76.7% vs. 50.0%, *p* = 0.030). This difference was no longer significant at 5 or 10 min, and all patients met discharge criteria (Aldrete ≥ 9) within 15 min (Table 4).

### 3.4. Pain, PONV, and Safety Outcomes

Postoperative pain was minimal in both groups throughout the PACU observation period, and no patient reported a VAS pain score > 2 in any assessment. Postoperative nausea and vomiting were uncommon and only nausea occurred in one patient in each group (Table 5). No cases of laryngospasm occurred in either group. Clinically relevant postoperative nasal bleeding requiring intervention (repeat nasal packing) occurred in 2/32 patients (6.3%) in the control group and in 0/30 patients (0%) in the remimazolam group. No clinically relevant respiratory complications within 24 h were recorded (Table 5).

Overall, remimazolam was associated with complete prevention of clinically relevant emergence agitation and with no cases of severe agitation requiring rescue sedation, without signals of delayed recovery or early adverse events.

## 4. Discussion

In this randomized trial, a single bolus of remimazolam given at the end of the surgery completely prevented clinically relevant emergence agitation after rhinologic surgery. Agitation (RASS ≥ 2 immediately after extubation) occurred in more than one-third of patients in the control group, while no cases were observed in the remimazolam group. Given the problematic nature of agitation in nasal surgery, this difference is clinically relevant.

Recent studies in adults have suggested that remimazolam may reduce emergence agitation after nasal surgery. Jang et al. reported no agitation events in the remimazolam group compared with a measurable incidence in patients receiving volatile anesthesia [14]. However, in their trial, remimazolam was used as the primary anesthetic technique rather than as a single emergence-phase intervention, which differs from the pragmatic approach evaluated in our study. In addition, Lu et al. demonstrated that a remimazolam bolus strategy can reduce emergence agitation in adults undergoing nasal surgery without delaying recovery [13]. While their findings support a beneficial effect of remimazolam in this setting, direct comparison with results of this study should be made with caution, given differences in study design, perioperative protocols, and agitation assessment. Absolute incidence rates of EA reported across trials vary widely, highlighting the heterogeneity of EA reporting in the literature [13].

Despite these differences, the direction of effect is consistent across studies and supports the concept that remimazolam administered around extubation can reduce clinically relevant agitation [13,14,15]. Importantly, our trial adds practical value by evaluating a single-bolus strategy within a standardized sevoflurane-based anesthetic technique, without requiring a switch to remimazolam-based anesthesia. In addition, our study used a triple-blind design and a strictly defined primary endpoint at extubation (RASS ≥ 2), which focuses on the clinically most relevant time window in rhinologic surgery.

The agitation rate in our control group (37.5%) was higher than in some trials, including the study by Jo et al., in which the incidence after nasal surgery was approximately 20% [5]. However, reported rates after nasal surgery vary substantially across the literature, largely depending on the definition of agitation and the timing of assessment [5,6].

In our protocol, we focused on the immediate post-extubation period and used a strict cut-off (RASS ≥ 2). We selected RASS ≥ 2 as the primary definition of clinically relevant agitation because it reflects overt restlessness and uncontrolled movements, which are particularly hazardous in rhinologic surgery immediately after extubation. This is arguably the most relevant time window in rhinologic surgery because sudden movements and confusion can lead to bleeding, airway events, or displacement of nasal packing [3,4]. In addition, clinically relevant postoperative nasal bleeding requiring repeat nasal packing occurred only in the control group, supporting the concern that uncontrolled movements during emergence may translate into bleeding-related complications in this setting. The relationship between bleeding and agitation is likely bidirectional. Severe agitation may displace nasal packing or traumatize the surgical site, while bleeding itself may exacerbate distress during recovery. Although the number of bleeding events was small, the absence of both agitation and bleeding in the remimazolam group suggests that effective control of emergence agitation may reduce secondary postoperative complications in rhinologic surgery.

The reduction in agitation was not associated with prolonged emergence or delayed early recovery. Extubation times were similar across groups; all patients met the Aldrete discharge criteria within 15 min, and no patient developed clinically meaningful oversedation (RASS ≤ −3). These findings suggest that the reduction in agitation was not attributable to delayed awakening. Moreover, agitation was assessed at extubation and repeatedly during the first 30 min in the PACU, and no late agitation was observed in either group, further arguing against simple masking of the agitation. Severe agitation (RASS ≥ 3) requiring rescue propofol sedation occurred in 6/32 control-group patients, while no patient in the remimazolam group required additional sedation. This is clinically relevant because it suggests that the bolus strategy not only reduced agitation but also minimized the need for additional sedative interventions during the most vulnerable phase of recovery.

The dose of 0.1 mg/kg was selected based on pharmacodynamic data indicating that this dose approximates the median effective dose (ED50) for loss of consciousness in adult patients while remaining well below the 95% effective dose (ED95) for respiratory depression, thereby providing an adequate safety margin during the emergence phase [16]. Remimazolam is currently approved mainly for procedural sedation in adults, where it is administered using fixed bolus dosing rather than weight-based (mg/kg) regimens.

Paradoxical agitation has been described with benzodiazepines, most commonly midazolam, but appears to be rare with remimazolam and has been reported mainly in isolated case reports rather than as a frequent adverse event in large clinical trials [17]. No paradoxical reactions were observed in our study population, possibly due to its extreme rarity, the exclusion of patients receiving psychiatric treatment, and the use of a single emergence-phase bolus.

Pain scores were uniformly low and comparable across groups, and no patient reported a VAS score > 2 during the PACU observation period. PONV was rare, occurring in one patient in each group. In line with our findings of an uncomplicated early recovery profile, remimazolam has also been associated with favorable early postoperative outcomes in other otolaryngologic procedures, including a reduced incidence of early postoperative nausea and vomiting compared with sevoflurane in tympanoplasty with mastoidectomy [18]. We also observed no laryngospasm and no clinically relevant respiratory complications within 24 h. Although the study was not powered for rare adverse events, these findings are reassuring.

Emergence agitation is likely multifactorial, and nasal surgery is a setting in which several triggers coexist, including airway irritation, rapid awakening, and the discomfort of nasal obstruction. In rhinologic surgery, nasal packing and postoperative nasal obstruction are recognized contributors to emergence agitation. The sensation of impaired nasal breathing may provoke anxiety, disorientation, and defensive movements during early recovery from anesthesia [2,5]. Individual anxiety traits may further modulate the subjective experience of postoperative nasal obstruction and potentially contribute to emergence agitation in susceptible patients [19]. Remimazolam may be particularly effective in this context because it provides short-lasting anxiolysis and sedation during the vulnerable emergence phase, while its rapid metabolism limits residual effects [10,11]. Specific phobias such as claustrophobia were not systematically assessed; however, no patient reported severe anxiety or panic symptoms during the preoperative evaluation.

From a health-economic perspective, anesthetic drug costs typically represent only a small proportion of the total cost of a surgical procedure. The addition of a single remimazolam bolus therefore contributes only marginally to the overall procedural cost, although it can significantly increase the cost of anesthetic medications themselves. Emergence agitation during recovery may substantially increase resource utilization through additional staff involvement, prolonged PACU monitoring, and management of bleeding or airway events requiring further intervention. This study was not designed as a formal cost-effectiveness analysis, but prevention of agitation-related complications may have important economic implications and warrants further evaluation.

Remimazolam can be reversed with flumazenil; however, routine administration is generally unnecessary due to its rapid metabolism and short context-sensitive half-time, which allow predictable and relatively fast spontaneous recovery. As with other benzodiazepines, flumazenil should nevertheless be readily available when remimazolam is used.

Remimazolam should not be used in patients with known hypersensitivity to remimazolam or other benzodiazepines or in those with unstable myasthenia gravis. Caution and dose adjustment may be warranted in older patients, in individuals with impaired cardiovascular or respiratory function, and in patients with higher perioperative risk (ASA physical status III–IV).

This study has several limitations. First, it was conducted at a single center with a relatively modest sample size, which limits generalizability and reduces the ability to detect uncommon adverse events. Second, emergence agitation was assessed only during the first 30 min in the PACU; therefore, later behavioral disturbances after discharge from the PACU could not be evaluated. Third, although our standardized anesthetic and analgesic protocol minimized confounding, the study was not designed to examine dose–response relationships or to compare remimazolam with other preventive agents, such as dexmedetomidine, ketamine, or opioid-based strategies. Although we did not observe any clinically relevant respiratory complications, the study was not powered to detect rare safety outcomes. Finally, our study compared remimazolam with a placebo and was not designed to evaluate superiority over other established preventive strategies, such as dexmedetomidine or opioid-based approaches.

Future multicenter studies with larger sample sizes are warranted to confirm these findings and to evaluate whether this simple bolus strategy improves clinically relevant outcomes such as postoperative complications, PACU resource use, and patient-reported recovery quality.

## 5. Conclusions

A single bolus of remimazolam 0.1 mg/kg given at the end of surgery prevented emergence agitation after rhinologic surgery without delaying early recovery.

## Figures and Tables

**Table 1:** Baseline characteristics of the study population.

Variable	Control (*n* = 32)	Remimazolam (*n* = 30)	*p*-Value
Sex (M/F)	18 (56.3)/14 (43.7)	12 (40.0)/18 (60.0)	0.201 *
Age (yr)	35.5 (25.0, 49.0)	35.0 (29.3, 44.8)	0.803 †
ASA PS I	12 (37.5)	14 (46.7)	0.465 *
Weight (kg)	83.7 ± 17.6	76.4 ± 15.5	0.090 ‡
Height (cm)	177.8 ± 8.4	175.4 ± 11.5	0.350 ‡
BMI (kg/m^2^)	26.31 ± 4.36	24.69 ± 3.50	0.114 ‡
Smoking	13 (40.6)	9 (30.0)	0.382 *
Hypothyroidism	2 (6.3)	4 (13.3)	0.672 §
Hypertension	7 (21.9)	2 (6.7)	0.149 §
COPD	2 (6.3)	0 (0.0)	0.492 §

Values are presented as mean ± SD for normally distributed variables, median (Q1, Q3) for non-normally distributed variables, or number (%). BMI: body mass index; ASA PS I: American Society of Anesthesiologists physical status I; COPD: chronic obstructive pulmonary disease. Statistical tests: * Chi-square test; † Mann–Whitney U test; ‡ Independent t-test; § Fisher’s exact test.

**Table 2:** Comparison of types of surgical procedures performed.

Variable	Control (*n* = 32)	Remimazolam (*n* = 30)
Septoplasty	15 (46.9)	17 (56.7)
Rhinoseptoplasty	10 (31.3)	4 (13.3)
FESS	3 (9.4)	2 (6.7)
FESS + septoplasty	3 (9.4)	4 (13.3)
FESS + rhinoseptoplasty	1 (3.1)	3 (10.0)

Values are presented as numbers (%). FESS: functional endoscopic sinus surgery.

**Table 3:** Comparison of operation, anesthesia, and extubation time.

Variable	Control (*n* = 32)	Remimazolam (*n* = 30)	*p*-Value
Surgery duration (min)	37.5 (28.8, 50.0)	37.5 (30.0, 58.8)	0.472 *
Anesthesia duration (min)	62.5 (55.0, 82.5)	62.5 (56.3, 83.8)	0.771 *
Extubation time (min)	13 (10.0, 17.3)	12 (10.0, 15.0)	0.280 *

Values are presented as median (Q1, Q3). * Mann–Whitney U test.

**Table 4:** Comparison of Aldrete scores at different time points.

Variable	Control (*n* = 32)	Remimazolam (*n* = 30)	*p*-Value
Aldrete score ≥ 9 at extubation	16 (50.0)	23 (76.7)	0.030 *
Aldrete score ≥ 9 at 5 min	23 (71.9)	26 (86.7)	0.153 *
Aldrete score ≥ 9 at 10 min	31 (96.9)	28 (93.3)	0.516 *

Values are present as numbers (%). * Chi-square test.

**Table 5:** PACU outcomes and safety.

Outcome	Control (*n* = 32)	Remimazolam (*n* = 30)
Emergence agitation (RASS ≥ 2) at extubation	12 (37.5)	0 (0.0)
Severe agitation (RASS ≥ 3) at any assessment	6 (18.8)	0 (0.0)
Rescue sedation with propofol required	6 (18.8)	0 (0.0)
Over-sedation (RASS ≤ −3) during PACU observation	0 (0.0)	0 (0.0)
VAS pain > 2 at any assessment	0 (0.0)	0 (0.0)
PONV	1 (3.1)	1 (3.3)
Laryngospasm	0 (0.0)	0 (0.0)
Clinically relevant nasal bleeding requiring intervention (repeat nasal packing)	2 (6.3)	0 (0.0)
Respiratory complications within 24 h *	0 (0.0)	0 (0.0)

Values are presented as numbers (%). PACU: post-anesthesia care unit; PONV: postoperative nausea and vomiting; RASS: Richmond Agitation–Sedation Scale; VAS: visual analog scale. * Defined as need for airway intervention, assisted ventilation, or peripheral oxygen saturation < 90%.

## Data Availability

The data presented in this study are available on request from the corresponding author. The data are not publicly available due to privacy restrictions and ethical considerations.

## Abbreviations

The following abbreviations are used in this manuscript:

EA	emergence agitation
PACU	post-anesthesia care unit
RASS	Richmond Agitation-Sedation Scale
VAS	visual analog scale
PONV	postoperative nausea and vomiting
ASA	American Society of Anesthesiologists
ASA PS	American Society of Anesthesiologists physical status
BIS	bispectral index
FESS	functional endoscopic sinus surgery
ARR	absolute risk reduction
CI	confidence interval
NNT	number needed to treat
SD	standard deviation
Q1, Q3	first and third quartile
ED50	median effective dose
ED95	95% effective dose
